# The Snf1-related protein kinases SnRK2.4 and SnRK2.10 are involved in maintenance of root system architecture during salt stress

**DOI:** 10.1111/j.1365-313X.2012.05089.x

**Published:** 2012-08-20

**Authors:** Fionn McLoughlin, Carlos S Galvan-Ampudia, Magdalena M Julkowska, Lotte Caarls, Dieuwertje van der Does, Christiane Laurière, Teun Munnik, Michel A Haring, Christa Testerink

**Affiliations:** 1 University of Amsterdam, Swammerdam Institute for Life Sciences, Section of Plant PhysiologyPostbus 94215, 1090GE Amsterdam, The Netherlands; 2 Centre National de la Recherche Scientifique, Institut des Sciences du VégétalUPR 2355, 1 Avenue de la Terrasse, Gif-sur-Yvette Cedex, France

**Keywords:** salinity stress, Snf1-related protein kinase, primary root growth, lateral root emergence, cellular membrane targeting, protein kinase activity, *Arabidopsis thaliana*

## Abstract

The sucrose non-fermenting-1-related protein kinase 2 (SnRK2) family represents a unique family of plant-specific protein kinases implicated in cellular signalling in response to osmotic stress. In our studies, we observed that two class 1 SnRK2 kinases, SnRK2.4 and SnRK2.10, are rapidly and transiently activated in Arabidopsis roots after exposure to salt. Under saline conditions, *snrk2.4* knockout mutants had a reduced primary root length, while *snrk2.10* mutants exhibited a reduction in the number of lateral roots. The reduced lateral root density was found to be a combinatory effect of a decrease in the number of lateral root primordia and an increase in the number of arrested lateral root primordia. The phenotypes were in agreement with the observed expression patterns of genomic yellow fluorescent protein (YFP) fusions of SnRK2.10 and -2.4, under control of their native promoter sequences. SnRK2.10 was found to be expressed in the vascular tissue at the base of a developing lateral root, whereas SnRK2.4 was expressed throughout the root, with higher expression in the vascular system. Salt stress triggered a rapid re-localization of SnRK2.4–YFP from the cytosol to punctate structures in root epidermal cells. Differential centrifugation experiments of isolated Arabidopsis root proteins confirmed recruitment of endogenous SnRK2.4/2.10 to membranes upon exposure to salt, supporting their observed binding affinity for the phospholipid phosphatidic acid. Together, our results reveal a role for SnRK2.4 and -2.10 in root growth and architecture in saline conditions.

## Introduction

Salinity stress is an increasing agricultural problem, limiting crop yield and plant productivity (Munns and Tester, 2008). Salinity causes instant osmotic stress, similar to drought and cold stress, and additionally a gradual ion accumulation, which is toxic to the plant. Plants respond very quickly to salt, i.e. by regulation of ion channels, generation of lipid signals, including phosphatidic acid (PA) and phosphoinositides, and by activation of protein kinase pathways (Boudsocq and Lauriere, 2005; Craig Plett and Moller, 2010; Galvan-Ampudia and Testerink, 2011; Hong *et al.*, 2010; Kulik *et al.*, 2011; Munnik and Vermeer, 2010; Testerink and Munnik, 2011; Zhu, 2002). One family of protein kinases that is activated upon salt treatment is the plant-specific sucrose non-fermenting-related kinase 2 family (SnRK2). Members of this protein kinase family have been identified in many different plant species, including *Zea mays* (maize) (Huai *et al.*, 2008), *Triticum aestivum L.* (wheat) (Anderberg and Walker-Simmons, 1992; Holappa and Walker-Simmons, 1995), *Glycine max* (soybean) (Monks *et al.*, 2001; Yoon *et al.*, 1997) and *Nicotiana tabacum* (tobacco) (Kelner *et al.*, 2004), and were shown to be activated upon salt and osmotic stress (Mikolajczyk *et al.*, 2000; Munnik *et al.*, 1999). In *Arabidopsis thaliana* and *Oryza sativa*, the SnRK2 family consists of 10 members of which, respectively, 9 out of 10 or all 10 members are activated upon osmotic stress (Boudsocq *et al.*, 2004; Kobayashi *et al.*, 2004).

Based on phylogeny, the SnRK2 family is divided into three classes (Kobayashi *et al.*, 2004), which differ in their activation in response to the phytohormone ABA. Arabidopsis class 3, comprising SnRK2.2 (SnRK2D), -2.3 (SnRK2I) and -2.6 (OST1; SnRK2E), are strongly activated in the presence of ABA, while class 2 members SnRK2.7 (SnRK2F) and -2.8 (SnRK2C) are activated to a lesser extent. In contrast, members of class 1, SnRK2.1 (SnRK2G), SnRK2.4 (SnRK2A), SnRK2.5 (SnRK2H), SnRK2.9 (SnRK2J) and SnRK2.10 (SnRK2B) are not activated in response to ABA (Boudsocq *et al.*, 2004; Boudsocq and Lauriere, 2005; Umezawa *et al.*, 2004).

Class 2 and 3 SnRK2s were shown to phosphorylate Ser/Thr residues in the R-X-X-S/T motif of the ABA responsive element-binding factor 2 (ABF2) and ABF4 transcription factors (Fujii *et al.*, 2007; Furihata *et al.*, 2006; Yoshida *et al.*, 2010). SnRK 2.7 and -2.8 are involved in drought signalling in an ABA-dependent way (Mizoguchi *et al.*, 2010). Using a phosphoproteomics approach, several targets of SnRK2.8 were identified that connect SnRK2.8 to metabolic processes (Shin *et al.*, 2007). SnRK2.6 was shown to play an important role in the regulation of stomatal conductance (Mustilli *et al.*, 2002; Yoshida *et al.*, 2002) by targeting the KAT1 potassium channel (Sato *et al.*, 2009), the slow-anion channel SLAC1 (Geiger *et al.*, 2009; Lee *et al.*, 2009) and AtrbohF NADPH oxidase (Sirichandra *et al.*, 2009). SnRK 2.2 and -2.3 are also activated by ABA, but control responses to ABA in seed germination, dormancy and seedling growth (Fujii *et al.*, 2007). The *snrk2.2/2.3/2.6* triple mutant is nearly insensitive to ABA, indicating redundancy between these genes (Fujii and Zhu, 2009; Fujita *et al.*, 2009; Nakashima *et al.*, 2009). The activity of SnRK2.6 is directly inhibited by the type 2C protein phosphatases ABI1, ABI2 and HAB1 and activation of SnRK2.6 occurs by de-repression of these phosphatases (Soon *et al.*, 2012; Umezawa *et al.*, 2009; Vlad *et al.*, 2009; Yoshida *et al.*, 2006), which in turn are contained by the soluble ABA receptors, PYR/PYL or RCAR in the presence of ABA (Ma *et al.*, 2009; Park *et al.*, 2009). The components PYR1, ABI1, SnRK2.6/2.2/2.3 and ABF2 were shown to be sufficient for ABA-induced gene expression, showing that class 3 SnRK2s act in the core ABA signalling pathway (Fujii *et al.*, 2009).

Compared with class 2 and 3 SnRK2 members, relatively little is known about the activation mechanism of class 1 members and their targets. Overexpression of the SnRK2.4 orthologue of wheat (TaSnRK2.4) in Arabidopsis has been shown to induce an increase in main root growth. Under drought conditions, overexpression lines had enhanced survival rates, which can be explained by their stronger water retention ability (Mao *et al.*, 2010). Using a semi-degenerate peptide array screen, SnRK2.10 has been demonstrated to target a preferential phosphorylation affinity motif that is conserved in the S-segment of dehydrins (Vlad *et al.*, 2008). Its orthologue in tobacco, NtOSAK, has been shown to directly interact with glyceraldehyde-3-phosphate dehydrogenase (GAPDH) (Wawer *et al.*, 2010), linking its mode of action to metabolic processes, similar to the SnRK2.8 in Arabidopsis (Shin *et al.*, 2007). A quadruple mutant of the ABA-independent class I SnRK2 members (*snrk2.1/2.4/2.5/2.10*) revealed elevated proline levels in response to osmotic stress (Fujii *et al.*, 2011).

To elucidate the function of the class 1 SnRK2s, we characterized the function of SnRK2.4 and -2.10, two previously described members (Boudsocq *et al.*, 2004; Testerink *et al.*, 2004) of this subgroup in Arabidopsis roots, and found that both were activated within 1 min of salt treatment. Subcellular localization studies and mutant analyses revealed that SnRK2.4 and -2.10 exert their function at different locations within the root. SnRK2.4 was found to be targeted to the membrane structures upon lateral root (LR) emergence or in response to salinity in epidermal cells. Moreover, knocking out either gene alters the growth and the architecture of Arabidopsis roots in saline conditions. Together, our data reveal distinct roles of SnRK2.4 and -2.10 in maintaining growth of both main roots and LRs under salinity stress.

## Results

### SnRK2.4 and -2.10 are among the fastest activated protein kinases in Arabidopsis roots upon salt stress

To investigate their possible role in salt stress signalling, activation of SnRK2.4 and -2.10 was studied in *A. thaliana* roots. Plants grown hydroponically for 28 days were transferred to control or saline medium, and kinase activity was monitored using an in-gel kinase assay on root protein extracts. To identify SnRK2.4 and -2.10 activation and to determine their roles in salt-related signalling, two independent T-DNA insertion lines were isolated for both kinases (*snrk2.4-1*, Salk_080588; *snrk2.4-2*, Salk_146522; *snrk2.10-1*, WiscDsLox233E9; *snrk2.10-2*, GABI_676G12) (Figure S1a in Supporting Information). Using an anti-SnRK2 (αSnRK2) antibody (Boudsocq *et al.*, 2004) these mutants were confirmed to be knock-outs at the protein level (Figure S1b).

**Figure 1:**
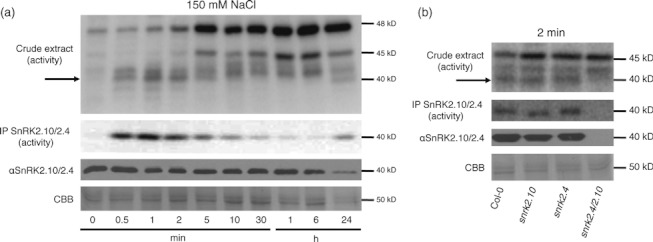
SnRK 2.4 and -2.10 are among the fastest activated protein kinases in *Arabidopsis thaliana* roots upon salt stress. (a) In-gel kinase assay of protein extracts from hydroponically grown roots exposed to 150 mm NaCl. In the upper panel (crude extract, activity) the activity of a number of kinases is detected. At 40 kDa the activation of SnRK2.4 and -2.10 is observed (indicated with an arrow). In the second panel, in-gel kinase activity is shown after immunoprecipitation (IP) with an antibody against SnRK2.4/2.10, confirming the identity of these bands to be SnRK2.4/2.10. In the third panel, the western blot analysis shows SnRK2.4 and -2.10 protein abundances at the different time-points and in the lower panel coomassie brilliant blue is shown as a loading control. (b) SnRK2.4 and 2.10 are responsible for the kinase activity observed at 40 kDs. The same approach as shown in (a) was conducted in the single and double mutant background after 2 min of 150 mm salt stress. All the protein kinase assays were performed with myelin basic protein immobilized in the gel as substrate.

Activation of SnRK2.4 and -2.10 was studied in roots of hydroponically grown plants that had been stressed by transfer to different salt concentrations ranging between 100 and 150 mm NaCl or 200 mm mannitol (Figure 1a and Figure S2b). Crude protein extracts were separated by SDS-PAGE with the generic protein kinase substrate myelin basic protein (MBP) immobilized in the gel. Myelin basic protein can be phosphorylated by several protein kinase families, including mitogen-activated protein (MAP) kinases (MAPKs) and SnRK2s (Boudsocq *et al.*, 2004; Droillard *et al.*, 2002; Munnik *et al.*, 1999), allowing visualization of the activity of the endogenous kinases. Two kinases with molecular weights of 45 and 48 kDa (presumably MAPKs) are activated between 2 min and 24 h (48 kDa) and 5 min and 6 h (45 kDa) in response to 150 mm NaCl (Figure 1a, upper panel). At the expected molecular weight of SnRK2.4 and -2.10 (40 kDa), a fast and transient activation of a protein kinase was observed (indicated by an arrow) between 30 sec and 5 min and the activation increased again after 24 h. To determine whether this protein kinase activity represented SnRK2.4, -2.10 or both, immunoprecipitation (IP) was performed with an antibody that recognizes both SnRK2.4 and -2.10 (Vlad *et al.*, 2010) and the protein kinase activity was analysed by an in-gel kinase assay (Figure 1a, second panel). In addition, kinase activation was investigated in the *snrk2.4*, *snrk2.10* and *snrk2.4/2.10* mutant backgrounds after a 2-min salt stress (Figure 1b). As shown in Figure 1a, the activation kinetics of the immunoprecipitated SnRK2s resembled the activation pattern observed at 40 kDa in the crude extract. Activation was very fast (<0.5 min), peaking at 1 min, and was rapidly repressed, but reappeared after 24 h. The identity of SnRK2.4 and -2.10 as the 40 kDa band in the crude extract was confirmed in the *snrk2.4/2.10* mutant background, where the 40 kDa band was completely absent after 2 min of salt stress. SnRK2.4 and -2.10 were activated to a similar degree since the 40 kDa kinase activity was similar in both single mutants (Figure 1b). Western blot analysis with the same antibody on the crude extract showed that there were no changes in protein abundance up to 6 h of salt treatment (Figure 1a, third panel). No kinase activation was observed when plants were transferred to control medium (Figure S2a). A similar activation pattern was observed when 200 mm mannitol was used (Figure S2b), indicating that SnRK2.4 and -2.10 are activated in Arabidopsis roots in response to salt and osmotic stress.

### SnRK2.4 and -2.10 play a role in maintaining root growth under saline conditions

**Figure 2:**
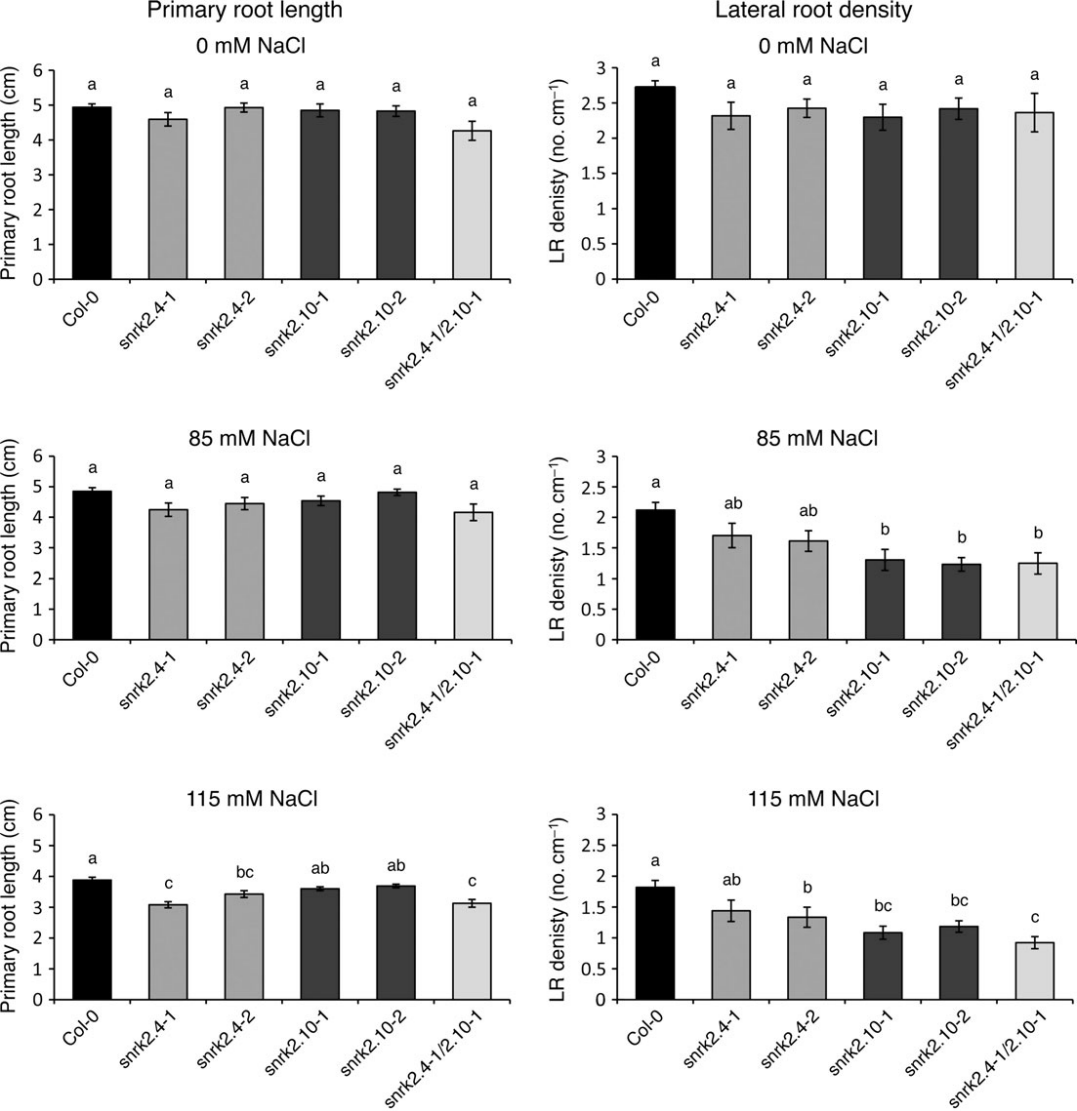
SnRK2.4 and -2.10 are involved in maintaining primary and lateral root growth, respectively, in saline conditions. *Arabidopsis thaliana* seeds of Col-0, *snrk2.4-1*, *snrk2.4-2*, *snrk2.10-1*, *snrk2.10-2* and *snrk2.4/2.10* were sown on agar plates containing ½ MS. The plants were grown vertically under an angle of 70°. After 4 days the seedlings were transferred either to control or plates supplemented with 85 or 115 mm salt. Plates were scanned and the primary root length (from the point of transfer to the root tip) and the lateral root density (LRD) were measured 8 days after the transfer (12-day-old seedlings) using image analysis software and counting of visual lateral roots. The primary root length (left panel) and the LRD (number of lateral roots/cm primary root, right panel) were averaged. The number of replicates varied between 20 and 30 replicates per line and concentration and seedlings were randomized over different plates. The phenotypes were confirmed in three independent studies. The error bars represent the standard error and significant differences were determined using Tukey-b and are indicated by letters (*P* ≤ 0.05).

In Arabidopsis, salinity been shown to cause changes in the root system architecture (Zhao *et al.*, 2011; Zolla *et al.*, 2010), allowing plants to optimize their growth under this condition. To assess whether the SnRK2 protein kinases play a role in this response, primary root length and lateral root density (LRD) were studied in the *snrk2.4* and -*2.10* mutants, in both control and saline conditions (85 or 115 mm NaCl) (Figure 2). To avoid any problems with sucrose affecting root growth through direct uptake via the leaves (Macgregor *et al.*, 2008), media were prepared without sucrose.

Under control conditions, there were no differences in primary root length when Col-0 wild type and all mutants were compared (Figure 2, left column). The addition of 85 mm NaCl to the growth medium did not change this, but when exposed to 115 mm NaCl, significant differences between the wild type and the *snrk2.4* mutant lines (*snrk2,4-1, snrk2.4-2*) appeared. A reduction of 35% in the primary root length was detected in *snrk2.4-1*, *snrk2.4-2* and the *snrk2.4-1/2.10-1* double mutant, whereas the wild type and the *snrk2.10* lines showed a reduction of only 20% when exposed to salt.

When studying the LRs (Figure 2, right column), no differences in LRD were observed between wild type and the mutants in control conditions. Col-0 showed a reduction in LRD of 20% at 85 mm NaCl in comparison to control conditions. In the *snrk2.10-1* and *snrk2.10-2* single mutants, a significantly greater reduction was observed, showing a reduction of close to 50% at 85 mm NaCl in comparison to control conditions. The double mutant again phenocopied the single mutants, also showing a 50% reduction in LRD. Similar results were obtained when exposing Col-0 and the mutant lines to 115 mm NaCl. These data show that both protein kinases are involved in maintaining root system architecture under saline conditions, where SnRK2.4 exerts its function predominantly in the primary root and SnRK2.10 in the LRs.

### The reduced number of lateral roots in *snrk2.10* and *2.4*/*2.10* mutants is primarily due to a reduction in the emergence of lateral roots

The reduction in LRD in the single *snrk2.10* and *snrk2.4/2.10* double mutant could either be due to a reduction in the number of lateral root primordia (LRP) or to a defect in their development. To investigate this in more detail, the developmental stages of all the primordia were studied.

**Figure 3:**
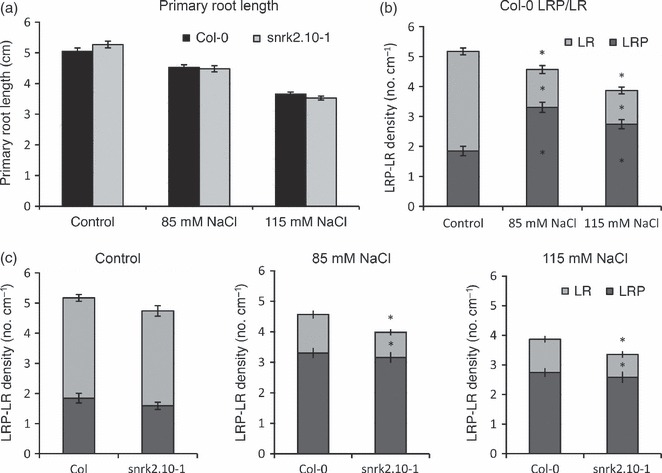
The *snrk2.10* knockout line exhibits a reduction in the number of lateral root primordia (LRP) in saline conditions. Col-0 and *snrk2.10* were grown and treated similarly as in Figure 2. (a) Primary root length of Col-0 and *snrk2.10* seedlings (*n* = 19–22). (b) Salt induces an increase in arrested LRP and a reduction in the total number of LRP. The lateral root density (LRD) of Col-0 was measured as in Figure 2. The total number of LRP was counted using a binocular after fixing and clearing the roots. The non-emerged LRP (dark bars), lateral roots (LR; light bars) and the total number of LRP (dark + light bars) were plotted per cm primary root. Asterisks in the dark and light bars represent significant differences in the density of non-emerged LRP and LRs, respectively. Asterisks above the bars indicate significant differences in the density of the total number of LRP. (c) The *snrk2.10* knock-out line shows a reduction in LRs and total LRP, but not in the non-emerged LRP. The number of replicates varied between 20 and 30 replicates per line and concentration and seedlings were randomized over different plates. The phenotypes were confirmed in three independent studies. The error bars represent the standard error and significant differences have been determined using a Student’s *t*-test (*P < 0.05).

As shown in Figure 3a, no difference in the primary root length was observed for the *snrk2.10* mutant compared with the Col-0 wild type under any of the conditions tested. For Col-0, the LRD and the non-emerged LRP density were measured at control conditions, and media supplemented with 85 and 115 mm NaCl (Figure 3b). In control conditions, 65% of the total lateral root primordia (LRP + LR) developed into a LR. When transferred to 85 mm NaCl, there was a 10% decrease in the total number of primordia (LRP + LR), but only 30% of the LRP developed into a LR. At the higher salt concentration (115 mm NaCl), the total number of primordia decreased by 25% compared with control conditions, but the percentage of LRP that developed into a LR was 30%, similar to that observed at 85 mm NaCl (Figure 3b).

In control conditions, no significant difference in either the LRD or LRP density was detected between Col-0 and *snrk2.10* (Figure 3c). At both 85 and 115 mm NaCl, the density of total lateral root primordia (LRP + LR) was less in *snrk2.10* than in the wild type. In addition to this reduction, a more pronounced effect was found in the percentage of LRP that developed into a LR (30% in Col-0, to 20% in the *snrk2.10* mutant background), which effectively accounted for the overall reduction of over 30% in the emerged LRD (Figure 3c). These results show that the reduction in LRD is partly due to a reduction in the total LRP density, but predominantly caused by a reduction in the percentage of LRP that developed into a LR, showing that SnRK2.10 plays a role in the development of LRP in saline conditions.

### Tissue-specific localization of SnRK2.4 and 2.10 in Arabidopsis roots

Their high homology at the amino acid sequence level indicated similar functions for the SnRK2.4 and -2.10 protein kinases. However, the knock-out mutants clearly showed different phenotypes in root system architecture in response to salt. To study their expression in roots, YFP-fusions of SnRK2.10 and -2.4 under the control of their native promoter sequence were constructed and transformed into their respective mutant backgrounds.

**Figure 4:**
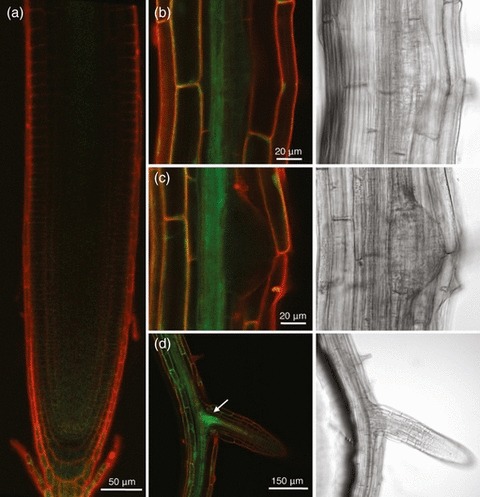
SnRK2.10 is expressed in the vascular tissue at the site of a lateral root. (a–d) pSnRK2.10::SnRK2.10-YFP was stably transformed into the *snrk2.10-2* mutant background. The mid-section of the primary root (a), lateral root primordia (LRP) in stage 3 (b), 6 (c) and emerged (d) were imaged. SnRK2.10–YFP is primarily expressed in the vascular tissue distal from the root tip. The arrow indicates the accumulation of SnRK2.10–YFP in the developing vascular tissue of an emerged lateral root. Prior to imaging, plants were exposed to propidium iodide for 2 min. Plants were grown vertically under an angle of 70° on agar plates containing ½ MS and 1% sugar for 7 days. SnRK2.10–YFP is shown in green and propidium iodide is shown in red. Wide-field images are depicted with their corresponding confocal images and all the pictures were taken with the same confocal settings.

In the primary root, SnRK2.10–YFP was not detectable in the root tip (Figure 4a) and was predominantly expressed in the vascular tissue in distal root tissue (Figure 4b–d). SnRK2.10–YFP was not detectable in the LRP during the initial stages of development in stages 3 and 6 (classification of LRP as described in Malamy and Benfey, 1997) (Figure 4b,c), but was highly expressed in the adjacent vascular tissue. Further from the root tip, SnRK2.10–YFP expression was higher in cortex cells (Figure 4d). In addition, SnRK2.10–YFP specifically accumulated in the developing vascular tissue of a newly emerged LR (Figure 4d).

**Figure 5:**
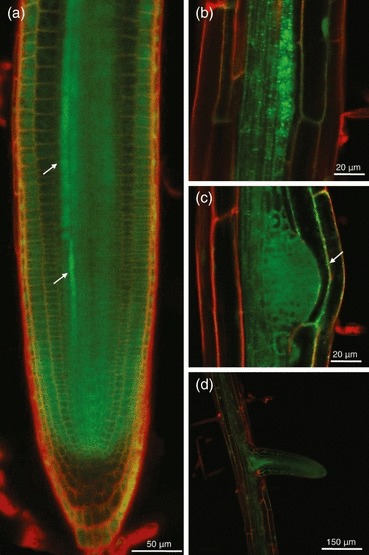
SnRK2.4 is expressed in cells surrounding an emerging lateral root primordium (LRP), and accumulates in punctate structures in these cells.(a–d) pSnRK2.4::SnRK2.4–YFP was stably transformed into the *snrk2.4-1* mutant. The mid-section of the primary root (a), LRP in stage 3 (b), stage 6 (c) and emerged (d) were imaged. (a) SnRK2.4–YFP accumulated in pericycle (indicated by the lower arrow), and the endodermis (indicated by the upper arrow). (b) At the site of a stage 3 LRP, SnRK2.4–YFP was highly expressed and accumulated in punctate structures. The expression in epidermal cells was lower compared than in the root tip. (c) Cortex cells surrounding the LRP contained higher levels SnRK2.4–YFP and similar punctate structures as observed in a stage 3 LRP (indicated by the arrow). (d) Accumulation in the developing vascular tissue of an emerged lateral root. SnRK2.4–YFP is shown in green and propidium iodide is shown in red. The growth conditions were the same and all the pictures were taken with the same confocal settings as used in Figure 4.

SnRK2.4–YFP was expressed in almost all cells proximal to the root tip of the primary root, excluding the columella cells. The highest accumulation of SnRK2.4–YFP was in pericycle cells (Figure 5a, lower arrow). Further distal from the root tip, SnRK2.4–YFP also accumulated in the endodermis (upper arrow), while its abundance in the adjacent cortex cells was reduced. Even further distal from the root tip, the expression in the epidermal cells was low (Figure 5b–d). Interestingly, at the site of a developing LRP (stage 3), SnRK2.4–YFP accumulated in punctate structures in the LRP (Figure 5b). At a later stage of LRP development (stage 6), SnRK2.4–YFP was present in the cytosol of all the LRP cells (Figure 5c). Remarkably, enhanced expression was observed in the cortex cells at the side of the developing LRP, which was not observed in the cortex cells on the other side. In addition, accumulation of SnRK2.4–YFP was observed in punctate structures in these cells (Figure 5c, indicated by the arrow). In the emerged LR, SnRK2.4–YFP was expressed at low levels in the developing vascular tissue, similar to the expression pattern of SnRK2.10–YFP (Figure 5d).

### Relocalization of SnRK2.4–YFP upon salt stress

**Figure 6:**
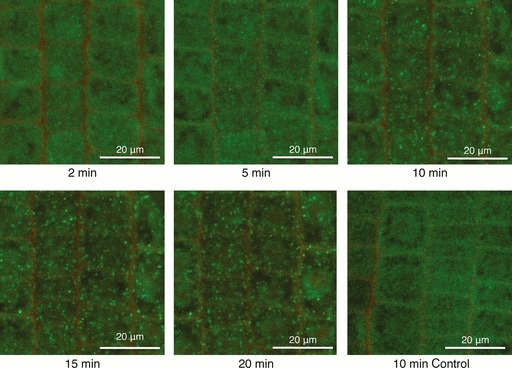
SnRK2.4–YFP accumulates in punctate structures in root epidermal cells upon salt stress. Seven-day-old *snrk2.4-1* pSnRK2.4::SnRK2.4–YFP seedlings were stained with propidium iodide for 2 min and subsequently placed either in control medium or medium containing 115 mm NaCl. The first image was taken 2 min after the saline medium was applied. Images were taken in epidermal cells approximately 150 μm above the root tip. In the lower right corner the same area is shown after 10 min of control treatment. SnRK2.4–YFP is shown in green and propidium iodide is shown in red. The growth conditions were the same and all the pictures were taken with the same confocal settings as used in Figure 4.

To investigate whether localization of SnRK2.4 or -2.10 would change in response to salt treatment, seedlings were treated with 115 mm NaCl. SnRK2.4–YFP relocalized from the cytosol to punctate structures within 5 min of salt application in epidermal cells (Figure 6, Video S1). Two minutes after the start of the treatment (first frame), SnRK2.4–YFP was still predominantly cytosolic, but after 15 min most of the SnRK2.4–YFP had moved from the cytosol and accumulated at unknown punctate structures. To confirm that the fusion protein was functional, western analysis and an in-gel kinase assay were performed on the SnRK2.4–YFP-expressing line, which showed that the fusion protein was intact and could be activated by salt treatment, similar to the endogenous SnRK2.4 protein kinase (Figure S3a,b). Moreover, an N-terminal GFP fusion of the SnRK2.4 protein could also be activated by salt (Figure S3a,b), and this fusion also relocalized to punctate structures upon salt treatment (Figure S3c), similar to SnRK2.4–YFP, ruling out any positional effect of the fluorescent protein tag on localization of the protein. SnRK2.10–YFP localization did not seem to be affected by salt treatment (up to 30 min).

A co-localization study with FM4-64, a lipophilic dye to label membranes, was performed to further investigate the nature of the accumulation of SnRK2.4–YFP (Figure S4). After treatment with the dye for 2 h prior to the 15-min salt stress treatment, co-localization occurred in some of the punctate structures that were closer to the membrane (indicated by the arrows), indicating that SnRK2.4–YFP is targeted to cellular membranes.

### Biochemical fractionation reveals membrane association of SnRK2.4 and/or SnRK2.10 upon salt stress

In order to further confirm the salt-induced recruitment to membranes, endogenous SnRK2.4/SnRK2.10 levels were analysed after subcellular fractionation in control and salt-stressed roots. This approach consisted of a number of different centrifugation steps, allowing the isolation of proteins that are associated with the membrane. A similar approach conducted on *Sorghum bicolour* was described earlier (Monreal *et al.*, 2010).

**Figure 7:**
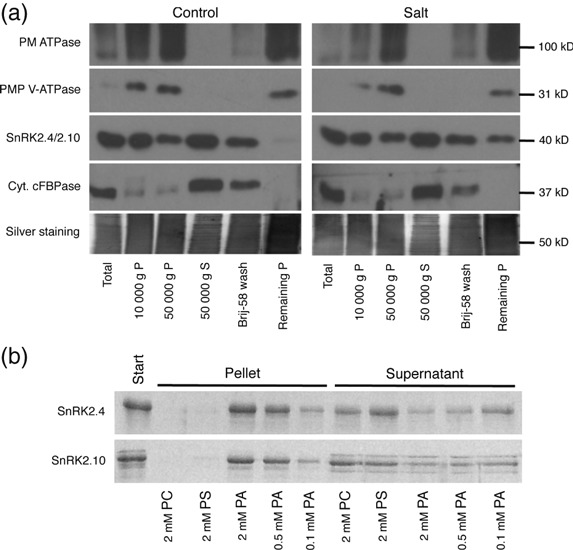
SnRK2.4/2.10 are targeted to cellular membranes during salt stress and bind phosphatidic acid (PA) *in vitro*. (a) Proteins from control or 150 mm NaCl-treated Arabidopsis roots were isolated and fractionated using sequential centrifugation steps. Pellet fractions are indicated with P and the supernatant fractions are indicated with S. Western blot analysis was performed on these fractions using antibodies against different compartment markers or SnRK2.4/2.10. Membranes (50 000 ***g*** pellet) were isolated and washed with Brij-58 to remove any cytosolic contaminants. From the upper to lower panel: plasma membrane (PM ATPase), peripheral vacuolar membrane (PMP V-ATPase ε subunit), SnRK2.4/2.10 and the cytosolic marker fructose-1,6-biphosphate (Cyt. cFBPase). In the lowest panel a silver stain is shown as a loading control. (b) Both SnRK2.4 and -2.10 bind liposomes containing PA. Purified glutathione *S*-transferase (GST)-tagged recombinant protein (1 μg per sample) was mixed with liposomes containing phosphatidylcholine (PC), PC/phosphatidylserine (PS) (1:1) or PC/PA (1:1). Lipid concentrations available for binding were 2 mm for PS and 2, 0.5 or 0.1 mm for PA. The start fraction represents the input, the pellet fraction represents the fraction that bound to the liposomes and the supernatant represents the remainder. Relative loading: start 50%, pellet 100%, supernatant 50%.

Different fractions were analysed by using antibodies against proteins that reside in different cellular compartments (Figure 7a). Protein concentrations of all fractions were normalized in order to determine relative alterations between fractions. The total, 50 000 ***g*** supernatant and the Brij-58 wash fraction mainly consisted of cytosolic proteins in both control and salt-stimulated samples. SnRK2.4/2.10 were highly abundant in these fractions, confirming their cytosolic localization. Both the 10 000 ***g*** (debris, intact organelles) and 50 000 ***g*** (microsomal membranes) pellet fractions contained cytosolic contamination, but contained mainly trans-membrane and peripheral membrane proteins. In comparison to the cytosolic marker, SnRK2.4/2.10 was enriched in these fractions indicating they were partially membrane bound in both control and saline conditions. When the cytosolic contaminants were removed by Brij-58 washing (Hardin *et al.*, 2004; Johansson *et al.*, 1995), a striking increase of SnRK2.4/2.10 was observed in the remaining pellet fraction of the salt-treated samples, but not in the control samples. The peripheral membrane marker, V-ATPase ε subunit, was equally present in this fraction in both samples, indicating that the same protein pools were isolated. The localization of SnRK2.4/2.10, in these fractionation studies indicated not only cytosolic but also membrane-associated localization in saline conditions, confirming the confocal microscopy observations and showing that SnRK2.4 is recruited to the membrane upon salt stress.

### Recombinant SnRK2.10 and SnRK2.4 both specifically interact with phosphatidic acid *in vitro*

Salinity stress is known to induce changes in the phospholipid composition of membranes in Arabidopsis, including the transient formation of PA (Bargmann *et al.*, 2009). SnRK2.10 was identified in a proteomic screen for PA targets and was shown to bind PA affinity beads (Testerink *et al.*, 2004). To test whether SnRK2.10 and its most related family member, SnRK2.4, directly bind to PA and to assess the specificity of lipid binding, recombinant GST–SnRK2.10 and GST–SnRK2.4 proteins purified from *Escherichia coli* were tested in a liposome binding assay (Figure 7b). Since liposomes consist of a lipid bilayer, interaction with a more naturally organized PA can be shown using this approach (Julkowska *et al.*, 2012; Testerink *et al.*, 2007). Both SnRK2.4 and -2.10 exhibited binding affinity for PA-containing liposomes in a concentration-dependent manner, while no binding could be detected for liposomes containing another anionic phospholipid, phosphatidylserine (PS), or control lipids consisting only of the structural phospholipid phosphatidylcholine (PC) (Figure 7b). Thus, SnRK2.10 and SnRK2.4 are able to bind PA directly and selectively *in vitro*, providing a possible molecular basis for the observed interaction of SnRK2.4 with cellular membranes upon exposure of roots to salt.

## Discussion

In plants, salinity stress activates several protein kinases that are implicated in salt acclimation signalling cascades (Galvan-Ampudia and Testerink, 2011; Kulik *et al.*, 2011). Most SnRK2-family members are activated in response to salt, and some of them were revealed to be important for ABA signalling and drought tolerance (classes 2 and 3). The role of the ABA-independent SnRK2s (class 1) is largely unknown (Kulik *et al.*, 2011). Here we took an approach to functionally characterize two of the class 1 SnRK2s. Although it has been suggested that all five class-1 SnRK2s (SnRK2.1, -2.4, -2.5, -2.9 and -2.10) act redundantly (Fujii *et al.*, 2011), we found that SnRK2.4 and -2.10 play distinct roles in maintaining root system architecture, affecting different parts of the root, largely corresponding to their respective expression patterns.

SnRK2.4 and -2.10 have previously been found to be activated by salt and hyperosmotic stress when transiently expressed in protoplasts (Boudsocq *et al.*, 2004). Here, we show their activation by salt and mannitol in Arabidopsis roots (Figure 1 and Figure S2b). Since activation is fast and transient, they most likely play a signalling role in the early responses to osmotic stress. Involvement of SnRK2.4/2.10 in abiotic stress signalling is consistent with the effect of overexpressing the SnRK2.4 orthologue in wheat (TaSnRK2.4), which increased the plant’s tolerance to drought, salt and cold stress (Mao *et al.*, 2010). Knocking out either SnRK2.4 or -2.10 in Arabidopsis affected root growth and architecture in saline conditions, but not in control conditions (Figure 2), showing that SnRK2.4 and -2.10 are involved in maintaining root growth during salt stress. As the SnRK2.4 orthologue in wheat has been described to be involved in the response to additional abiotic stress stimuli (Mao *et al.*, 2010) and SnRK2 class 1 kinases are generally activated in response to osmotic stress, it is likely that the function of these kinases is not restricted to salt stress signalling and might play a broader role in abiotic stress signalling, including mechanical stress occurring during LR development.

Although SnRK2.4 and -2.10 are highly homologous at the amino acid level (91%), different phenotypes were observed under saline conditions for each mutant; the absence of SnRK2.4 resulted in a reduction of primary root growth, while the absence of SnRK2.10 resulted primarily in a reduction of the lateral root. The expression patterns of both kinases are consistent with the observed phenotypes in the knockout mutants (Figures 4 and 5). This indicates that although their knockout phenotypes are different, cellular functions of both kinases could be similar.

SnRK2.10 was shown to be important in the development from a LRP to a LR (Figure 3). The LRP were classified into the developmental stages 1–7, emerged or LR (Figure S5a) as described in Malamy and Benfey (1997). In Col-0, there is an increase in non-emerged LRP in saline conditions (Figure S5b). This was due to an increase in the number of LRP arrested in stages 5 and 6 (Figure S5b). The observed reduction in lateral root formation in Col-0 in salt stress in consistent with most published studies (Deak and Malamy, 2005; Galvan-Ampudia and Testerink, 2011). Stage 5 and 6 LRP were not overrepresented in the *snrk2.10* mutant (Figure S5c), but LRs were rather arrested at the emergence stage, indicating that SnRK2.10 plays a role during or right after the emergence of a newly formed lateral root. This observation is consistent with the expression pattern of SnRK2.10 in the root, since it accumulated specifically in the developing vascular tissue of an emerged LR and was hardly present in the earlier LRP developmental stages (Figure 4). Taken together these data indicate that SnRK2.10 plays a role in the emergence and further outgrowth of lateral roots during salt stress.

Although, the expression pattern of SnRK2.4 at the site of a developing LR also points to a function in LR development, no LR phenotype was observed in the *snrk2.4* mutants, possibly due to redundancy with another SnRK2 class 1 member. In the cortex cells, where the LRP is applying mechanical pressure to its neighbouring cells, high expression of SnRK2.4, but not -2.10, was observed (Figure 5c). Here, SnRK2.4–YFP accumulated in punctate structures, similarly to the first stages of LRP development (Figure 5b) or in response to salt stress (Figure 6). Since all cells that contain these punctate structures are exposed to mechanical stress, this could be a plausible cause of the relocalization. In accordance, salt has been shown to induce swelling of cortex cells after 24 h, which would result in mechanical strain (Dinneny *et al.*, 2008). Although the observed SnRK2.4 relocalization is much faster (i.e. within 5–10 min), it is possible that initial changes in the cortex cells that lead to swelling could trigger the localization of SnRK2.4 to punctate structures.

Co-localization with the lipophilic dye FM4-64 suggested that SnRK2.4–YFP is associated with intracellular membranes (Figure S4). This was confirmed through a cellular fractionation of root extracts (Figure 7a), where only after being exposed to salt stress was SnRK2.4/2.10 found to be associated with membrane fractions. As SnRK2.10 was not expressed in the epidermal cells in which SnRK2.4 was found to relocalize, and the biochemical approach cannot distinguish the individual isoforms, it is unknown whether SnRK2.10 could also relocalize, similar to SnRK2.4.

Membrane association of both isoforms is consistent with the identification of SnRK2.10 in a proteomics screen for binding to the phospholipid PA, reported earlier (Testerink *et al.*, 2004), and the specific binding of both SnRK2.10 and SnRK2.4 to PA-containing liposomes observed here (Figure 7b). Phosphatidic acid rapidly accumulates in response to several stress conditions and is an important signalling lipid in all eukaryotes, affecting the localization and function of a diverse set of target proteins (Testerink and Munnik, 2011), among which are several plant protein kinases. These include the Arabidopsis Constitutive Triple Response 1 (CTR1) (Testerink *et al.*, 2007), phosphoinositide-dependent kinase 1 (PDK1) (Anthony *et al.*, 2004), mitogen-activated protein kinase 6 (MPK6) (Yu *et al.*, 2010) and a *Zea mays* calcium-dependent protein kinase (CDPK) (Klimecka *et al.*, 2011). Relocalization of SnRK2.4 might thus be mediated through an interaction with PA, which could possibly affect interaction of class 1 SnRK2 isoforms with their direct phosphorylation targets. No *in vivo* phosphorylation targets have been described for SnRK2.4 and -2.10. Yet, a conserved S-segment of stress-related dehydrin protein family members was found to be preferentially phosphorylated by SnRK2.10 *in vitro* (Close, 1996; Vlad *et al.*, 2008). Dehydrins are implicated in drought, cold and salt stress and have binding affinity to anionic, negatively charged lipids through their conserved K-segment (Close, 1996; Koag *et al.*, 2003, 2009). One Arabidopsis dehydrin, Lti30, was further characterized and was reported to interact electrostatically with several anionic lipids, including PA. The interaction of Lti30 with the membrane depends on its phosphorylation status, and alters the fluidity of the membrane (Eriksson *et al.*, 2011). Since SnRK2.4 binds PA and is recruited to the membrane in response to salt, PA might act as a docking station, possibly spatially facilitating dehydrin phosphorylation and docking to the membrane.

Another candidate target of SnRK2.4 is the glycolytic enzyme GAPDH, which is an interaction partner of NtOSAK, a SnRK2.4 orthologue in *N. tabacum* (Wawer *et al.*, 2010). We have identified GAPDH to be a PA-binding protein using PA-beads (F. McLoughlin S.A. Arisz, H.L. Dekker, G. Kramer, M.A. Haring, T. Munnik, C. Testerink, in preparation). Post-translational modification has been shown to influence the interaction of GAPDH with the surface of mitochondria and lipid–protein interactions are proposed to be necessary for its stabilization (Graham *et al.*, 2007).

**Figure 8:**
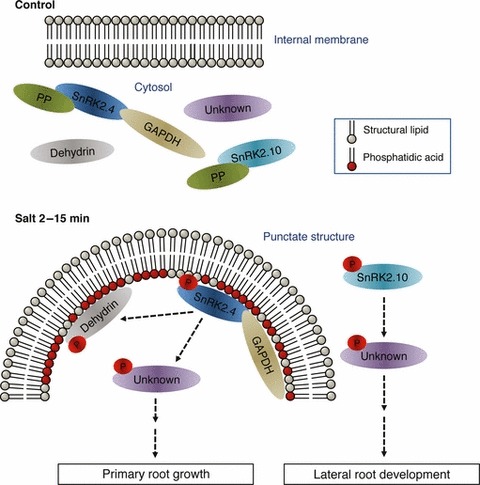
A model of the activation, transient localization, cellular partners and the role of SnRK2.4 and SnRK2.10 in root system architecture in saline conditions. SnRK2.4 and -2.10 are rapidly and transiently activated in response to salt stress, possibly due to a de-repression of an unknown phosphatase. After 15 min both SnRK2.4 and -2.10 are inactive again. It is unknown whether relocalization to punctate structures or membrane binding is required for the activation or its role in the maintenance of the root system architecture in saline conditions, or whether these reflect an attenuating mechanism. After 5–15 min of salt stress, SnRK2.4 is present in punctate structures, while for SnRK2.10 no accumulation in punctate structures was observed, hence it is drawn as cytosolic in this figure. It is possible SnRK2.10 would also relocalize as the expression of SnRK2.10 is low in the outer cell layers of the root where the relocalization of SnRK2.4 in response to salt is most pronounced, and both protein kinases were shown to bind phosphatidic acid. Little is known about the downstream targets of SnRK2.4 and -2.10. Unknown downstream effectors of SnRK2.4 and -2.10 are hypothesized to mediate the effects on primary and lateral root growth. Abbreviations used: SnRK, sucrose non-fermenting-1-related protein kinase; PP, protein phosphatase; GAPDH, glyceraldehyde-3-phosphate dehydrogenase. Structural lipids are represented as grey circles and phosphatidic acid as red circles.

In this work, physiological function, localization and cellular dynamics of SnRK2.4 and -2.10 have been uncovered. Our findings suggest that class 1 SnRK2s play a role in linking the perception of salt stress to modulation of root growth and development. In Figure 8, these findings and their possible implications for root growth under saline conditions are summarized in a working model. Since root system architecture in both control and saline conditions is highly controlled by phytohormones including auxin, cytokinin, ABA and ethylene (Fukaki and Tasaka, 2009; Galvan-Ampudia and Testerink, 2011), a possible role for SnRK2.4 and -2.10 in these signaling cascades should be investigated. In addition, identification and/or verification of phosphorylation targets of SnRK2.4 and -2.10 could reveal the molecular basis of the SnRK2.4 and -2.10 phenotypes in root growth and development.

## Experimental Procedures

### Isolation of homozygous T-DNA insertion lines and generation of plants expressing YFP and GFP-fusions

Homozygous lines were selected by PCR using gene-specific primers for two independent T-DNA insertion lines (Alonso *et al.*, 2003) for both SnRK2.4 (*At1g10940*) and SnRK2.10 (*At1g60940*) using; *snrk2.4-1* (Salk_080588), *snrk2.4-2* (Salk_146522), *snrk2.10-1* (WiscDsLox233E9) and *snrk2.10-2* (GABI_676G12). Primers are listed in Table S1. The *snrk2.4-1* and *2.10-1* mutants were crossed to obtain a double mutant. Western blot analysis with an αSnRK2 antibody (Boudsocq *et al.*, 2004) of crude protein extracts of 8-day-old seedlings was performed to confirm absence of the proteins. The SnRK2 antibody was used as the primary antibody.

The genomic promoter and coding region of *SnRK2.4* (chromosome 1, 3659208–3656052) were amplified using the attB1SnRK2.4 and attB2SnRK2.4 primers that contained Gateway adapters. The same was conducted for *SnRK2.10* (chromosome 1, 22444519–22439397) using the attB1SnRK2.10 and attB2SnRK2.10 primers (Table S1). The fragments were recombined into pDONR207 using BP clonase (Invitrogen, http://www.invitrogen.com/). All the fragments were verified by sequencing. Subsequently the fragments were recombined into the expression vector pGreen0179 PL Gateway YFP HA using LR clonase (Invitrogen). These constructs were transformed via the *Agrobacterium tumefaciens* strain GV3103 in their respective mutant backgrounds, *snrk2.4-1* and *snrk2.10-2*, through floral dip transformation (Clough and Bent, 1998). Several primary transformants were selected using 30 μg ml^−1^ hygromycin and the plants were allowed to self. Recombinant proteins of the correct size were confirmed by western blot analysis using a αGFP polyclonal antibody (Molecular Probes, http://www.invitrogen.com/site/us/en/home/brands/Molecular-Probes.html).

For the construction of the GST fusions and 35S::GFP-overexpression lines, *SnRK2.10* and *SnRK2.4* cDNAs were amplified using the specific primer sets R4F and R4R for *SnRK2.10*, and R5F and R5R for *SnRK2.4.* Subsequently, *SnRK2.10* and *SnRK2.4* PCR products were amplified with generic AttB1-F and AttB2-R primers to generate attB recombination sites, and were recombined into pDONR207. The resulting entry vectors were used in LR clonase recombination reactions with pDEST15, to generate GST-SnRK2.10 and GST-SnRK2.4 fusion constructs for expression in *E. coli*, or with pK7WGF2, to generate 35S:GFP-SnRK2.10 and 35S:GFP-SnRK2.4 constructs for expression in plants.

### In-gel kinase assay

*Arabidopsis thaliana* plants were grown hydroponically (http://www.araponics.com/) for 4 weeks under short-day conditions (light/dark 10 h/14 h, 21°C/70% humidity) with a weekly change of growth medium using the Flora series (GHE, http://gb.eurohydro.com/company.html). Twenty-four hours prior to stimulation, plants were transferred to smaller containers (three plants per container). For each sample, three plants were treated by transferring the plants to containers containing control or medium supplemented with salt or mannitol.

Proteins were extracted from ground root tissue using one volume of extraction buffer [50 mm 2-amino-2-(hydroxymethyl)-1,3-propanediol (TRIS)/HCl pH 7.5, 5 mm EDTA, 5 mm EGTA, 2 mm DTT, 25 mm NaF, 1 mm Na_3_VO_4_, 50 mmβ-glycerophosphate, 1× complete protease inhibitor cocktail (Boehringer Ingelheim, http://www.boehringer-ingelheim.com/)] and a 10 min 10 000 ***g*** centrifugation step. Protein concentration was determined using Bradford (Bio-rad, Veenendaal, the Netherlands).

For immunoprecipitation, 500 μg of proteins were combined with 25 μl αSnRK2.4/2.10 serum (Vlad *et al.*, 2010) and IP buffer [50 mm TRIS/HCl pH 7.5, 150 mm NaCl, 1× complete protease inhibitor cocktail (Boehringer Ingelheim), 0.2% (v/v) tergitol-type NP-40] was added to a total volume of 500 μl. Samples were gently rotated for 3 h at 4°C. Fifty microlitres of a 50% Protein G (GE Healthcare, http://www3.gehealthcare.com/en/Global_Gateway) slurry in IP buffer was added and incubated overnight at 4°C while gently rotating. The samples were spun at 10 000 ***g*** for 2 min and washed in IP buffer three times. The supernatant was completely removed after the last washing step and the proteins were eluted using 40 μl 1× sample buffer (60 mm TRIS/HCl, 2% SDS, 5%β-mercaptoethanol, 10% glycerol, 0.02% bromophenol blue).

Proteins were separated on a 12% polyacrylamide gel containing 0.2 mg ml^−1^ MBP (Upstate, http://www.millipore.com/company/cp1/redirect-ab). Gels were washed three times for 30 min in wash buffer [25 mm TRIS/HCl pH 7.5, 500 μm DTT, 100 μm Na_3_VO_4_, 5 mm NaF, 500 μg ml^−1^ BSA, 0.1% (v/v) Triton X-100] at 18°C (RT) and additionally washed twice for 30 min and then overnight using regeneration buffer (25 mm TRIS/HCl pH 7.5, 1 mm DTT, 100 μm Na_3_VO_4_, 5 mm NaF) at 4°C. Gels are washed once at RT for 30 min in reaction buffer (25 mm TRIS/HCl pH 7.5, 2 mm EGTA, 12 mm MgCl_2,_ 1 mm DTT, 100 μm Na_3_VO_4_) and then incubated in reaction buffer supplemented with 25 μm of cold ATP and 50 μCi ^32^P γ-ATP for 1 h. Gels were washed six times over a period of 5 h in stop buffer [1% (w/v) Na_2_H_2_P_2_O_7_, 5% (v/v) trichloric acid]. Gels were dried and the signal was visualized by exposing the gels to a phosphoimage screen (Amersham Biosciences, http://www.gelifesciences.com/) and read by a Storm (Molecular Dynamics, http://www.gelifesciences.com/).

### Root growth assays

Seeds were surface sterilized in a desiccator in the presence of 100 ml household bleach supplemented with 3 ml HCl for 3 h. Seeds were sown on square plates containing ½ MS and 1% Daishin agarose (Duchefa, http://www.duchefa.com/), pH 5.8 (KOH) and vernalized at 4°C for 48 h. Plants were grown under long-day conditions (21°C, 70% humidity, 16-h/8-h light/dark) for 4 days until the plants were either transferred to control or plates supplemented with salt. At 8 days after transfer the plates were scanned and roots measured using Object Image software. To visualize the stages of primordia, roots were fixed and cleared as described in (Dubrovsky *et al.*, 2009). Primordia were studied and classified using an Olympus BH-2 microscope.

### Confocal microscopy

Plants were grown on square plates containing ½ MS (Duchefa), 1% Daishin agar and 1% sucrose (pH 5.8, KOH). Plants were either grown on these plates for 7 days or transferred after 3 days to pre-fixed microscope slides and grown for an additional 4 days between the slides. For the salt treatment, control media were substituted with media containing the corresponding amount of salt. The fluorophores were either: excited with argon 514 nm; emission YFP, 525-555 nm; or propidium iodide (600–650 nm). In the case of Figure S4: excitation is by argon 488 nm, emission by YFP, 525–555 nm; excitation by argon 596 nm, emission FM4-64 570–620. Pictures were taken with a Nikon A1 with a 20× water lens (http://www.nikon.com/). Pictures were processed using ImageJ.

### Fractionation

Col-0 was grown similarly as for the in-gel kinase assay. Thirty millilitres of root material was harvested of either control or salt stressed roots (approximately 160 plants per treatment). Fractionation was essentially performed as described in Monreal *et al.* (2010). Crude protein was extracted by grinding the tissue in liquid nitrogen and incubating it in protein extraction buffer [50 mm TRIS pH 7.5, 300 mm sucrose, 5 mm EDTA, 5 mm EGTA, 2 mm DTT, 1× complete protease inhibitors (Boehringer Ingelheim)] for 10 min. Samples were filtered through Miracloth and centrifuged at 1500 ***g***, 2 min, 10 000 ***g***, 10 min six times where the pellets were stored at −20°C for analysis. Membranes were isolated by spinning for 2 h at 50 000 ***g***. The membranes were washed by homogenizing the pellet using protein extraction buffer with an additional 0.1% Brij-58 (Sigma-Aldrich, http://www.sigma-aldrich) (Johansson *et al.*, 1995). Membranes were again spun down at 50 000 ***g*** for 1 h. The pellet fraction was washed twice again in protein extraction buffer as described above and the final pellet was dissolved in 1 ml of protein extraction buffer. The antibodies raised against specific protein markers were obtained from http://www.agrisera.com: PM ATPase (At2g18960), PerM V-ATPase (At4g11150), Cyt. cFBPase (At1G43670). The SnRK2.4/2.10 specific antibody was described in Vlad *et al.* (2010). Silver staining was conducted as a loading control.

### Lipid-protein-binding assays

The constructs harbouring *GST-SnRK2.10* and *GST-SnRK2.4* were transformed to *E. coli* strain BL21-A1 and expression of the fusion proteins was induced using 0.2% arabinose for 3 h at 22°C. The GST fusion proteins were purified using affinity chromatography on glutathione agarose as described before (Testerink *et al.*, 2007). Bound protein was eluted from the glutathione agarose resin using elution buffer containing 20 mm reduced glutathione in 50 mm TRIS-HCl pH 8.0. Liposome-binding assays were performed as described in Julkowska *et al.* (2012). Per sample, 1000 ng protein was incubated with liposomes of varying lipid compositions as indicated for 1 h, after which the liposomes were spun down and washed once. All phospholipids were obtained from Avanti Polar Lipids. Start, pellet and supernatant fractions were loaded on SDS-PAGE and proteins were detected using colloidal coomassie brilliant blue staining.

## SUPPORTING INFORMATION

**Figure S1.** Isolation and confirmation of *snrk2.4* and *2.10* knockout mutants and the generation of the SnRK2.4–YFP and SnRK2.10–YFP recombinant lines.

**Figure S2.** SnRK2.4 and −2.10 are activated when exposed to 100 mM NaCl as to 200 mM mannitol, but not when transferred to control medium.

**Figure S3.** The SnRK2.4–YFP fusion is a functional protein kinase and re-localization of SnRK2.4 to punctate structures in response to saline conditions occurs independently of the location of the fluorophore.

**Figure S4.** The SnRK2.4–YFP fusion partially co-localizes with the endocytotic marker FM4-64.

**Figure S5.** The distribution of lateral root primordia developmental stages is altered in *snrk2.10*.

**Video S1.** SnRK2.4 accumulates in punctate structures in response to salt stress.

**Table S1.** Primers used.

please note: As a service to our authors and readers, this journal provides supporting information supplied by the authors. Such materials are peer-reviewed and may be re-organized for online delivery, but are not copy-edited or typeset. Technical support issues arising from supporting information (other than missing files) should be addressed to the authors.
