# Supplementary material for: The Snf1-related protein kinases SnRK2.4 and SnRK2.10 are involved in maintenance of root system architecture during salt stress
Source: Plant J. 2012 Aug 20;72(3):436–49. doi: 10.1111/j.1365-313X.2012.05089.x (PMC3533798; doi:10.1111/j.1365-313X.2012.05089.x)
Supplement: Supplementary file 7 [file tpj0072-0436-SD6.doc]

**Supporting Information legends**

**Figure S1.** Isolation and confirmation of SnRK2.4 and 2.10 knockout mutants and the generation of the SnRK2.4-YFP and SnRK2.10-YFP recombinant lines. (a) A schematic overview of the coding region of SnRK2.4 and 2.10. The triangles indicate the locations of the T-DNA insertions with the according names and the insertion line identities. (b) SnRK2.4 and 2.10 have a similar molecular weight. Proteins from seedlings were extracted and separated on a 12% polyacrylamide gel and subjected to western blot analysis using the αSnRK2 antibody. SnRK2.4 was represented by the more intense upper band and SnRK2.10 by the less intense lower band. In the single mutants either of the proteins were gone corresponding to the mutant and in the double mutant both proteins were gone. (c) The promoter and genomic sequence of SnRK2.4 and 2.10 fused to YFP were transformed to their corresponding mutant background. The proteins of 8-day-old seedlings were isolated and separated using SDS-PAGE. Recombinant proteins were detected through western blot analysis using a polyclonal αGFP antibody. Both SnRK2.4-YFP and SnRK2.10 YFP show a recombinant protein at 70 kD, which corresponds to the calculated size. In the lower panel a loading control is displayed using CBB.

**Figure S2.** SnRK2.4 and 2.10 are activated when exposed to 100mM NaCl as to 200 mM mannitol but not when transferred to control medium. Hydroponically grown plants were transferred to either control or medium supplemented with 100mM NaCl, 200mM mannitol or 150 mM NaCl. (a) Transfer to control medium did not induce any visible kinase activation. Plants were transferred to control (C) or medium containing 150 mM NaCl (S) for 2 minutes or 24 hours and kinase activity was determined to exclude an effect of transferring the plants to fresh medium. The panels are constructed in the same way as described in Fig. 1. (b) The activation in response to 100 and 150 mM and 200 mM mannitol is displayed in the upper three panels. The arrows indicate the activity of SnRK2.4 and 2.10. To confirm that the activity is due to SnRK2.4 and 2.10, the activity was also determined in the *snrk2.4/2.10* double mutant two minutes after the transfer to saline medium. No activity could be detected in the *snrk2.4/2.10* mutants at 40 kD. 100 mM NaCl and 200 mM mannitol resulted in similar activation patterns. When treated with 150 mM NaCl there is also a transient activation, but the total activation is higher. In addition, the activation of SnRK2.4/2.10 after 24 hours only occurs when exposed to 150 mM NaCl. The lower three panels show a western blot analysis using an antibody against SnRK2.4/2.10 to determine their abundances in each time-course. All kinase assays were performed with MBP as the substrate.

**Figure S3.** The distribution of LRP developmental stages is altered in *snrk2.10*. (a) A visual representation of the developmental stages of LRP with their corresponding nomenclature. (b) In mild saline conditions less LRP develop into a LR and more LRP are arrested in stage 5 and 6. All the primordia on the primary root were classified and the distribution of the developmental stages over the total length of the primary root was determined. **c.** Less LRP developed into a LR in *snrk2.10* compared to Col-0 in saline conditions. These data are obtained from the same experiment as shown in fig. 3 and the distribution of all the roots was determined and averaged. The error bars represent the standard error.

**Figure S4.** The SnRK2.4-YFP fusion is a functional protein kinase and re-localization of SnRK2.4 to punctate structures in response to saline conditions occurs independently of the location of the fluorophore. (a) Both GFP-SnRK2.4 (N-terminally fused GFP) and SnRK2.4-YFP (C-terminally fused YFP) are detected by an αSnRK2 antibody. The GFP-SnRK2.4 was under the control of a 35S::promoter, therefore only 20% of the amount of protein in comparison to the other samples was loaded on SDS-PAGE. CBB is shown as a loading control. (b) GFP-SnRK2.4 and SnRK2.4-YFP are both activated in response to salt. Hydroponically grown *Arabidopsis* roots were treated with 150 mM NaCl. The crude protein extract was separated on SDS-PAGE and an in-gel kinase assay was conducted using MBP as substrate. **c.** 35s::GFP-SnRK2.4 (N-terminally fused GFP) was transformed to Col-0. The roots were exposed to 150mM NaCl for 2 and 5 minutes. GFP-SnRK2.4 also accumulates in punctate structures showing that the localization occurs independently of the localization of the fluorophore.

**Figure S5.** The SnRK2.4-YFP fusion partially co-localizes with the endocytotic marker FM4-64. 7 day-old seedlings were pre-treated with FM4-64 for 2 hours in advance before exposing them to saline conditions for 10 minutes. SnRK2.4-YFP is shown in the green and FM4-64 in the red channel. SnRK2.4-YFP in punctate structures close to the plasma membrane co-localized with the FM4-64 dye (indicated by the arrows). The co-localization was less striking further away from the plasma membrane.

**Video S1.** SnRK2.4 accumulates in punctate structures in response to salt stress. Epidermal cells were imaged just after exposure to 115 mM NaCl. Frames of this video are shown in Fig. 6. The video shows the cellular localization of SnRK2.4-YFP. One frame per minute is shown, between 2 and 25 minutes after applying 115 mM NaCl.

**Table S1.** Primers used.
