# Supplementary material for: The Snf1-related protein kinases SnRK2.4 and SnRK2.10 are involved in maintenance of root system architecture during salt stress
Source: Plant J. 2012 Aug 20;72(3):436–49. doi: 10.1111/j.1365-313X.2012.05089.x (PMC3533798; doi:10.1111/j.1365-313X.2012.05089.x)
Supplement: Supplementary file 8 [file tpj0072-0436-SD7.docx]

**Table S1.** Primers used.

| Name | Sequence |
| --- | --- |
| SnRK 2.4-1 F | CTCTGTTCTCGTGTTTGCCT |
| SnRK 2.4-1 R | GGACTCATTCTCATCATCAACC |
| SnRK 2.4-2 F | CTCGAAGAGAATATGATGGC |
| SnRK 2.4-2 R | GTTTTCCTGAAGTTCTTGGG |
| SnRK 2.10-1 F | CTCACTCCAAAGCGTCGAAGAG |
| SnRK 2.10-1 R | CTCTGCATCTTCCTCCTTGC |
| SnRK 2.10-2 F | TTCTTTATGCATGGCTCAAGG |
| SnRK 2.10-2 R | GTAGCGCTGGAAGATTCAGTG |
| lBb1.3 (Salk) | ATTTTGCCGATTTCGGAAC |
| WiscDsLox BP | AACGTCCGCAATGTGTTATTAAGTTGTC |
| GABI BP | GGGCTACACTGAATTGGTAGCTC |
| attB1SnRK2.4 | GGGGACAAGTTTGTACAAAAAAGCAGGCTTACAAAAACAACGATAAGGC |
| attB2SnRK2.4 | GGGGACCACTTTGTACAAGAAAGCTGGGTAACTTATTCTCACTTCTCC |
| attB1SnRK2.10 | GGGGACAAGTTTGTACAAAAAAGCAGGCTTAGATTATGGCTTGTGGAAATGG |
| attB2SnRK2.10 | GGGGACCACTTTGTACAAGAAAGCTGGGTAACTGACTCGGACTTCTCC |
| R5F | AAA AAG CAG GCT CAA TGG ACA AGT ACG AGC TGG TG |
| R5R | AGA AAG CTG GGT ATC AAC TTA TTC TCA CTT CTC C |
| R4F | AAA AAG CAG GCT CAA TGG ACA AGT ACG AGC TTG TT |
| R4R | AGA AAG CTG GGT TTT AAC TGA CTC GGA CTT CTC CC |
| AttB1-F | GGG GAC AAG TTT GTA CAA AAA AGC AGG CT |
| AttB2-R | GGG GAC CAC TTT GTA CAA GAA AGC TGG GT |
